# Simultaneous Mapping of Vasculature, Hypoxia, and Proliferation Using Dynamic Susceptibility Contrast MRI, ^18^F-FMISO PET, and ^18^F-FLT PET in Relation to Contrast Enhancement in Newly Diagnosed Glioblastoma

**DOI:** 10.2967/jnumed.120.249524

**Published:** 2021-10

**Authors:** Solène Collet, Jean-Sébastien Guillamo, David Hassanein Berro, Ararat Chakhoyan, Jean-Marc Constans, Emmanuèle Lechapt-Zalcman, Jean-Michel Derlon, Mathieu Hatt, Dimitris Visvikis, Stéphane Guillouet, Cécile Perrio, Myriam Bernaudin, Samuel Valable

**Affiliations:** 1 Normandie University, UNICAEN, CEA, CNRS, ISTCT/CERVOxy Group, GIP Cyceron, Caen, France;; 2 Radiophysics Department, Centre François Baclesse, Caen, France;; 3 Department of Neurology, CHU de Caen, Caen, France;; 4 Department of Neurology, CHU de Nimes, Nimes, France;; 5 Department of Neurosurgery, CHU de Caen, Caen, France;; 6 Department of Neuroradiology, CHU de Caen, Caen, France;; 7 Department of Pathology, CHU de Caen, Caen, France;; 8 Department of Neuropathology, GHU Paris Psychiatry and Neuroscience, Paris, France;; 9 LaTIM, INSERM, UMR 1101, University of Brest, Brest, France; and; 10 Normandie University, UNICAEN, CEA, CNRS, ISTCT/LDM-TEP Group, GIP Cyceron, Caen, France

**Keywords:** proliferation, vasculature, hypoxia, MRI, PET, glioblastoma

## Abstract

Conventional MRI plays a key role in the management of patients with high-grade glioma, but multiparametric MRI and PET tracers could provide further information to better characterize tumor metabolism and heterogeneity by identifying regions having a high risk of recurrence. In this study, we focused on proliferation, hypervascularization, and hypoxia, all factors considered indicative of poor prognosis. They were assessed by measuring uptake of ^18^F-3'-deoxy-3'-^18^F-fluorothymidine (^18^F-FLT), relative cerebral blood volume (rCBV) maps, and uptake of ^18^F-fluoromisonidazole (^18^F-FMISO), respectively. For each modality, the volumes and high-uptake subvolumes (hot spots) were semiautomatically segmented and compared with the contrast enhancement (CE) volume on T1-weighted gadolinium-enhanced (T1w-Gd) images, commonly used in the management of patients with glioblastoma. **Methods:** Dynamic susceptibility contrast-enhanced MRI (31 patients), ^18^F-FLT PET (20 patients), or ^18^F-FMISO PET (20 patients), for a total of 31 patients, was performed on preoperative glioblastoma patients. Volumes and hot spots were segmented on SUV maps for ^18^F-FLT PET (using the fuzzy locally adaptive bayesian algorithm) and ^18^F-FMISO PET (using a mean contralateral image + 3.3 SDs) and on rCBV maps (using a mean contralateral image + 1.96 SDs) for dynamic susceptibility contrast-enhanced MRI and overlaid on T1w-Gd images. For each modality, the percentages of the peripheral volumes and the peripheral hot spots outside the CE volume were calculated. **Results:** All tumors showed highly proliferated, hypervascularized, and hypoxic regions. The images also showed pronounced heterogeneity of both tracers regarding their uptake and rCBV maps, within each individual patient. Overlaid volumes on T1w-Gd images showed that some proliferative, hypervascularized, and hypoxic regions extended beyond the CE volume but with marked differences between patients. The ranges of peripheral volume outside the CE volume were 1.6%–155.5%, 1.5%–89.5%, and 3.1%–78.0% for ^18^F-FLT, rCBV, and ^18^F-FMISO, respectively. All patients had hyperproliferative hot spots outside the CE volume, whereas hypervascularized and hypoxic hot spots were detected mainly within the enhancing region. **Conclusion:** Spatial analysis of multiparametric maps with segmented volumes and hot spots provides valuable information to optimize the management and treatment of patients with glioblastoma.

## Visual Abstract

**Figure:**
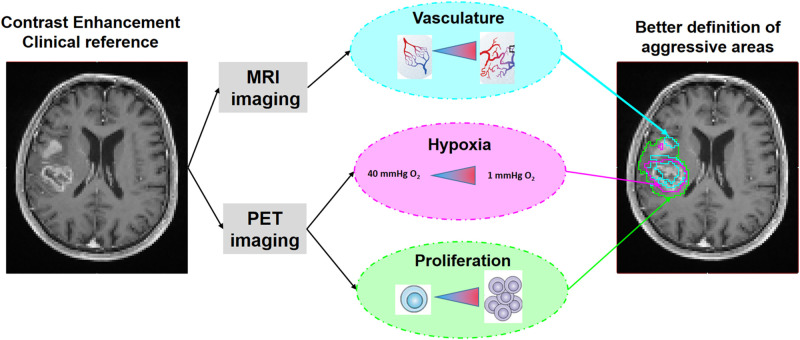

Despite the use of aggressive treatments (1), glioblastoma remains one of the deadliest human cancers, being characterized by a 5-y survival of 6.8% (2). Glioblastomas are highly heterogeneous tumors characterized by a strong interpatient heterogeneity at both the molecular (3,4) and the macroscopic levels. More importantly, glioblastomas are also characterized by a pronounced intratumoral heterogeneity (5), which is macroscopically visible on conventional MRI as regions of necrosis and contrast enhancement (CE) (6) and has been associated with a large range of response to therapies (7).

Among the various pathophysiologic parameters that may influence patient survival, proliferation and invasion are 2 key parameters considered to be predictive of patient survival (8). Interestingly, the dynamic interactions among tumor cells, the vasculature, and hypoxia are also considered a key feature that affects tumor growth (9–11).

Although various work has addressed the spatial relationship between pairs of parameters (10,12–17), the concomitant and quantitative measurement of these 3 parameters remains challenging and has been performed only on histologic specimens (9), which do not allow for an overall view of the entire 3-dimensional (3D) tumor volume.

Until now, conventional MRI with the so-called CE area remains the most used imaging modality to characterize glioblastoma and guide treatment. However, multiparametric imaging is most appropriate to assess biologic tumor heterogeneity and, more specifically, to quantitatively analyze these 3 compartments together.

Specific imaging markers of tumor activity have emerged recently, providing additional information to further characterize the tumor and its environment (18,19). In the field of neurooncology, these markers include those derived from multiparametric MRI, such as perfusion, diffusion, and MR spectroscopy. For PET imaging, the radiotracers that have emerged as most pertinent for this tumor type are those reflecting cell proliferation, such as ^18^F-3'-deoxy-3'-^18^F-fluorothymidine (^18^F-FLT) (13,20,21); those that trace amino acids, such as ^11^C-methionine, *O*-(2-^18^F-fluoroethyl)-l-tyrosine or 3,4-dihydroxy-6-^18^F-fluoro-l-phenylalanine (22); and those that can specifically differentiate true tumor boundaries from equivocal lesions on the basis of the degree of hypoxia, such as ^18^F-fluoromisonidazole (^18^F-FMISO) (10,12,23).

However, to the best of our knowledge, a spatial analysis of these 3 parameters—that is, proliferation, hypervascularization, and hypoxia—has never been reported using noninvasive imaging for newly diagnosed glioblastoma, and only a few studies have performed such a characterization in other tumor locations (24,25). Interestingly, the most proliferative, vascularized, and hypoxic subvolumes (i.e., hot spots) could also represent regions at high risk of relapse, and consequently, their identification is of real interest to overcome resistance to therapies such as surgery or radiation therapy.

Therefore, in this study, we aimed to spatially evaluate the volumes and hot-spot subvolumes of proliferation, hypervascularization, and hypoxia by using ^18^F-FLT PET, relative cerebral blood volume (rCBV) MRI, and ^18^F-FMISO PET, respectively, relative to CE volume in preoperative glioblastoma patients.

## MATERIALS AND METHODS

### Patients

Patients with de novo glioblastoma were included from 2 prospective clinical trials (“FLT” study and “HypOnco” study) funded by Institut National du Cancer and approved by the local ethics committee and Agence Française de Sécurité Sanitaire des Produits de Santé (French Agency for the Safety of Health Products) agreement (ClinicalTrials.gov identifiers NCT00850278 and NCT01200134). Thirty-one patients were included at the Caen University Hospital on the basis of the inclusion criteria: presenting with histopathologically proven grade IV gliomas based on World Health Organization criteria, being eligible in the final analysis with MR and PET imaging modalities, having an age of at least 18 y, having a Karnofsky Performance Status of at least 50%, having a normal blood cell count and normal biologic hepatic function, and providing written informed consent to voluntary participation in research. The patients underwent ^18^F-FLT PET (*n* = 20) or ^18^F-FMISO PET (*n* = 20) and multiparametric MRI (*n* = 31) (Table 1) within the same week and before surgery. Thereafter, the patients underwent surgery, resection, or biopsy depending on the location of the tumor. The specimens were histopathologically evaluated by an experienced neuropathologist, and only patients with an established diagnosis of glioblastoma were analyzed.

**TABLE 1:** Flowchart of Study

	FLT study		
		HypOnco study	
Patient no.	^18^F-FLT PET	rCBV MRI	^18^F-FMISO PET
1	✔	✔	
2	✔	✔	
3	✔	✔	
4	✔	✔	
5	✔	✔	
6	✔	✔	
7	✔	✔	
8	✔	✔	
9	✔	✔	
10	✔	✔	✔
11	✔	✔	✔
12	✔	✔	
13	✔	✔	
14	✔	✔	✔
15		✔	✔
16	✔	✔	✔
17	✔	✔	✔
18	✔	✔	✔
19	✔	✔	✔
20	✔	✔	✔
21	✔	✔	✔
22		✔	✔
23		✔	✔
24		✔	✔
25		✔	✔
26		✔	✔
27		✔	✔
28		✔	✔
29		✔	✔
30		✔	✔
31		✔	✔

HypOnco = Hypoxia in Brain Tumors; ✔ = patients were followed in the “FLT study” by ^18^F-FLT PET along with MRI or in the “HypOnco study” by ^18^F-FMISO along with MRI. Some patients were followed in the 2 studies.

### Image Acquisition

MRI was performed on a 1.5-T GE Healthcare Signa HDXt, version 15.0. After scout-view and coronal T2-weighted imaging, an axial fluid-attenuated inversion recovery (FLAIR) sequence was performed (24 slices; slice spacing, 5.5 mm; pixel resolution, 0.47 × 0.47 mm; repetition time/echo time, 9,602/150 ms). For dynamic susceptibility contrast-enhanced MRI, a dynamic gradient-echo T2*-weighted echoplanar imaging sequence was used (14 slices; 35 repetitions; slice spacing, 7 mm; pixel resolution, 2.19 × 2.19 mm; repetition time/echo time, 2,280/60 ms) to track a 0.1 mmol/kg bolus of gadolinium‐DOTA (Dotarem; Guerbet). An injection delay of 20 s was applied to obtain an accurate estimate of the baseline signal intensity before arrival of the contrast agent, and the acquisition lasted 1 min 20 s.

Immediately thereafter, a 3D T1-weighted gadolinium-enhanced (T1w-Gd) sequence (124 slices; slice spacing, 1.5 mm; pixel resolution, 1.01 × 1.01 mm; repetition time/echo time, 17/3 ms) was performed to evaluate the CE.

^18^F-FLT and ^18^F-FMISO were both produced by the LDM-TEP (Laboratoire de Développement Méthodologique en TEP) group of ISTCT (Imaging and Therapeutical Strategies in Cerebral and Tumoral Pathologies) and GIP Cyceron (a biomedical imaging platform facility) and synthesized as previously described (12,13,26). Data within a brain-focused field of view were acquired on 2 consecutive days 40 min (^18^F-FLT) and 2 h (^18^F-FMISO) after the intravenous injection of 5 MBq/kg (both tracers) and lasted 10 min (^18^F-FLT, to match a clinically feasible approach) and 20 min (^18^F-FMISO). Acquisitions were performed on a GE Healthcare Discovery VCT 64 PET/CT scanner (Cyceron platform). The CT-based attenuation-corrected PET images were reconstructed with an ordered-subsets expectation maximization 2-dimensional algorithm (9 subsets and 2 iterations) and filtered in 3 dimensions with a Butterworth filter on a 1.95 × 1.95 × 3.27 mm voxel size. SUVs (g/mL) were calculated using the measured concentration in tissue (counts, kBq/mL) divided by the injected activity (kBq/g of body weight).

### Image Analysis

MR image analysis was performed with in-house macros based on ImageJ software (27). PET analyses were performed with PMOD software, version 3.1.

rCBV maps were computed using dynamic susceptibility contrast-enhanced MRI. Variations of the T2* signal in the tissue were calculated with in-house macros based on ImageJ software as follows: ΔR_2_*(t) = −1·ln(S(t)/S_0_)/echo time, where R_2_ is the transverse relaxation rate (corresponding to 1/T2), expressed in msec-1 and t is time, ln = natural logarithm, S(t) is signal intensity over time, and S_0_ is signal intensity before contrast agent injection. Then, cerebral blood volume (CBV) maps were generated by integrating the area under the γ-variate–fitted curves to avoid an effect of recirculation (28). Images were then normalized by dividing CBV maps by the mean value of the normal-appearing contralateral side to obtain rCBV maps.

#### Coregistration

rCBV maps, FLAIR, 3D T1w-Gd, and ^18^F-FMISO PET images were coregistered with trilinear interpolation, rigid matching, and normalized mutual information on ^18^F-FLT PET images (PMOD software, version 3.1).

#### Volume Segmentation

In the present study, we had to tune the segmentation for each imaging modality since none of the various methods we used was considered pertinent enough for the 3 imaging modalities, which differed in term of contrast-to-tumor ratio and signal intensity. Also, we paid attention to the accuracy of the segmentation modality for all patients in 1 imaging modality. When 2 methods were almost the same, we retained the most restrictive one to avoid any overinterpretation of our results.

#### Volume Segmentation for ^18^F-FLT PET

The visual inspection easily enabled us to eliminate 40% of SUV_max_, which underestimates volume, whereas a mean contralateral image (Mean Contra) + 3.3 SDs overestimates volume (Supplemental Fig. 1; supplemental materials are available at http://jnm.snmjournals.org). A semiautomated fuzzy locally adaptive bayesian (FLAB) algorithm previously validated for ^18^F-FLT PET images was exploited, using 2 or 3 classes (29–33).

#### Volume Segmentation for ^18^F-FMISO PET

We considered FLAB, a standard 1.2 tissue-to-blood segmentation, 40% of SUV_max_, and Mean Contra + 3.3 SDs, and compared them with each other quantitatively and visually.

As exemplified in Supplemental Fig. 2, the standard 1-to-2 tissue-to-blood segmentation failed for some patients. For 40% of SUV_max_, it was visually striking that an overestimation of hypoxic volumes occurred. We then performed either FLAB segmentation or a semiautomated segmentation using a statistical approached based on the Mean Contra + 3.3 SDs (34). As compared with the tissue-to-blood segmentation approach, we believe this segmentation could be very well suited in the routine situation because it does not require drawing and processing of radioactive blood. Thus, with the exception of a few pixels removed by hiding with FLAIR hypersignal, no further manual intervention was necessary, making this technique particularly well suited. This statistical approach, Mean Contra + 3.3 SDs, seems suitable for a tracer such as ^18^F-FMISO with an average uptake and a large SD in the healthy brain parenchyma and a poor tumor–to–contralateral-tissue contrast.

FLAB and Mean Contra + 3.3 SDs led to very similar results (Supplemental Fig. 2). In some cases, FLAB provided slightly larger hypoxic volumes than Mean Contra + 3.3 SDs; we retained the less permissive strategy.

#### Volume Segmentation for CBV

The CBV segmentation relates to the ^18^F-FMISO situation. Because a volume of blood is present in healthy brain tissue, the statistical approach resulted in accurate segmentation compared with the visual approach using the naked eye. We used an already-published methodology (35) assuming a threshold of 2 or 3 times the signal of the normal-appearing white matter. We also compared this first methodology with a contralateral region of interest (ROI) composed of both gray and white matter and using a threshold of Mean Contra + 1.96 SDs. For both methods, only the region included in the FLAIR region was retained. The 2 methods provided very similar results, but Mean Contra + 1.96 SDs was less permissive (Supplemental Fig. 3).

#### Hot-Spot Segmentation

For each modality, the hot-spot area was defined as the 95th percentile of histogram distribution in the 3D ROI defined by the FLAIR hypersignal. This definition was designed to include all voxels that may extend to the CE ROI. All segmented areas were then used as 3D ROIs for further studies.

#### Peripheral Volume and Hot-Spot Calculation

After the segmentation process, for each modality and each patient we defined a peripheral volume and a peripheral hot-spot subvolume as the percentage of the ROI of the modality of interest that is outside the volume of CE. This volume was calculated using a Boolean operation in the following equations.

$$\text{Peripheral volume (\%) = }\frac{\text{ROI of the volume of the modality NOT ROI of CE}}{\text{ROI of CE}}\;\times \text{ 100}$$

$$\text{Peripheral hot spot (\%) = }\frac{\text{ROI of the hot spot of the modality NOT ROI of CE }}{\text{ROI of CE}}\;\times \text{ 100}$$

## RESULTS

**FIGURE 1.**
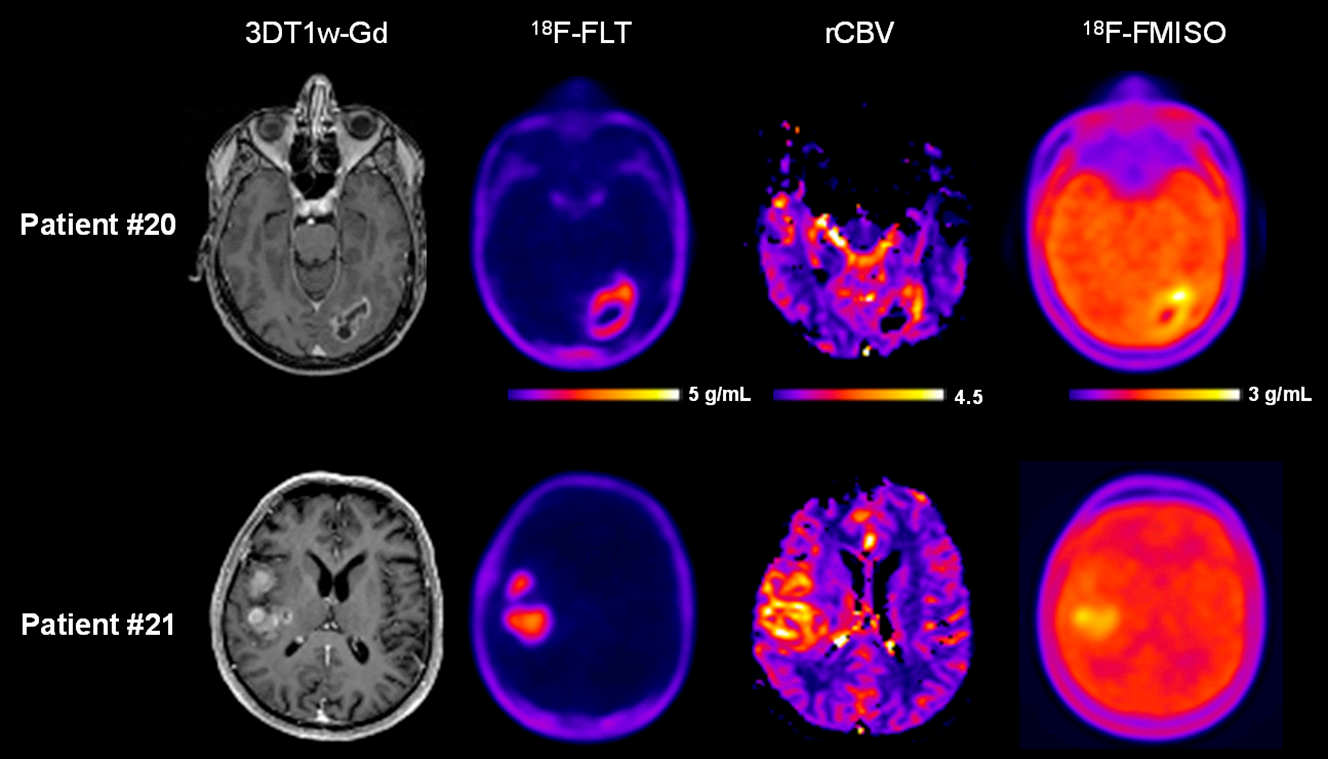
Multimodal imaging of 2 glioblastoma patients with 3D T1w-Gd, ^18^F-FLT PET, rCBV MRI, and ^18^F-FMISO PET.

All tumors were confirmed to be a glioblastoma by the pathologist and exhibited a marked CE on 3D T1w-Gd images, elevated rCBV, and pronounced ^18^F-FLT and ^18^F-FMISO uptake. Figure 1 shows representative examples of multimodal imaging of 2 glioblastoma patients, including 3D T1w-Gd MR images, ^18^F-FLT PET images, rCBV maps, and ^18^F-FMISO PET images. On the basis of visual inspection by an expert in PET imaging, a marked intratumoral heterogeneity of tracer uptake on both PET images was observed. Since the CE is the main target of treatments (surgery or radiation therapy), we then paid attention to the spatial relationship between each modality and the CE region.

### Analyses of the Peripheral Volume Outside the CE Region

**FIGURE 2.**
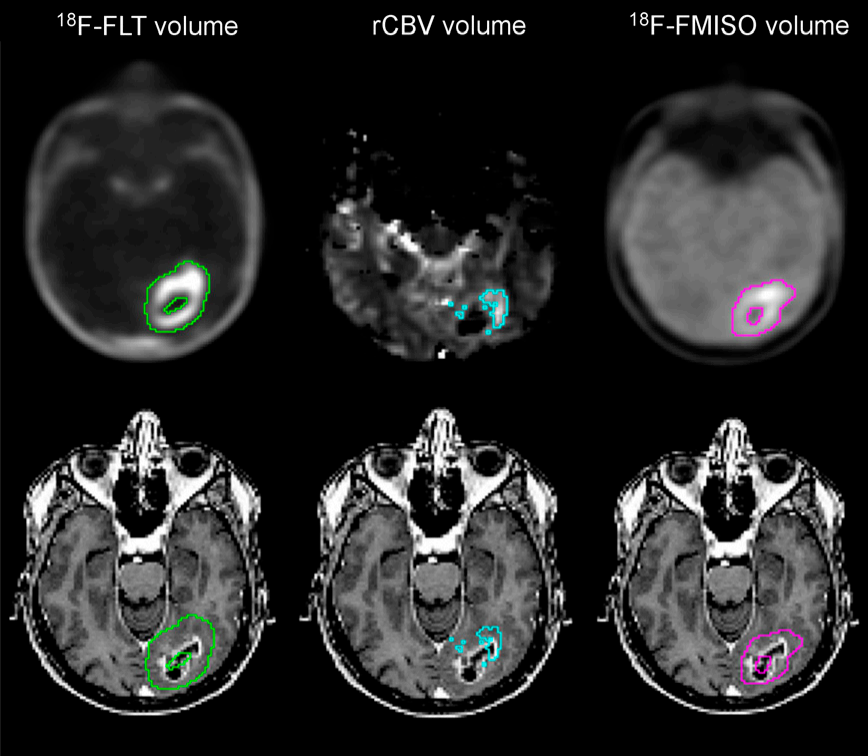
Example of proliferative volume in green, hypervascularized volume in blue, and hypoxic volume in pink on segmented (top) and overlaid (bottom) 3D T1w-Gd MR images.

**FIGURE 3.**
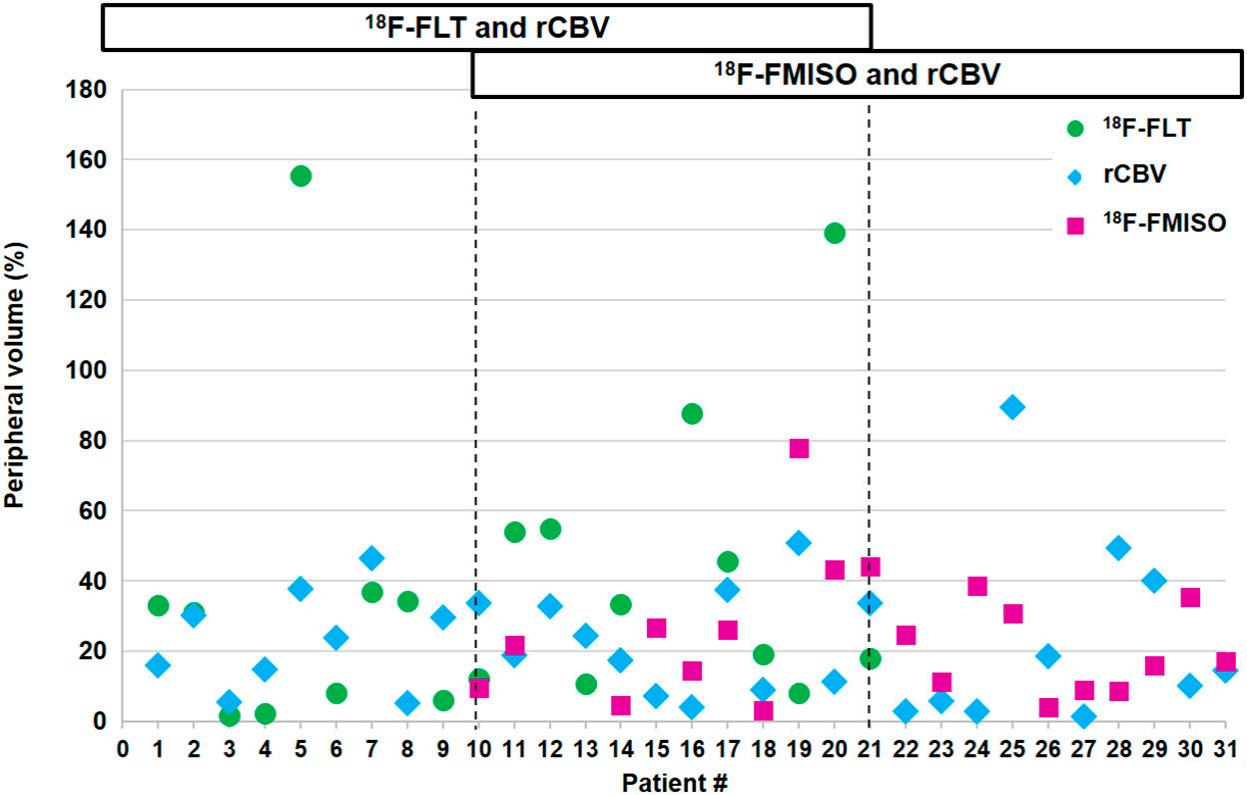
Percentage of peripheral volume of ^18^F-FLT, rCBV, and ^18^F-FMISO outside CE volume.

Thresholded regions of proliferation with ^18^F-FLT, of hypervascularization with rCBV, and of hypoxia with ^18^F-FMISO (Fig. 2, top) were overlaid on the T1w-Gd images (Fig. 2, bottom). Figure 2 and the calculated peripheral volume (Fig. 3, patient 20) illustrated that the volume of ^18^F-FLT uptake extended far from the CE area (139%). A similar situation also occurred for ^18^F-FMISO uptake but was less pronounced, with the peripheral volume being 43% whereas for CBV only 11% of the segmented area extended into the nonenhancing area. A representative example of the 3 modalities’ segmentation overlayed on the T1w-Gd image is provided (Supplemental Fig. 4).

The calculated peripheral volume outside the CE volume for each patient (Fig. 3) clearly demonstrated that extension of metabolic areas beyond the CE volume was highly variable. The ranges of peripheral volumes for ^18^F-FLT, rCBV, and ^18^F-FMISO were, respectively, 1.6%–155.5%, 1.5%–89.5%, and 3.1%–78.0%. More precisely, over the 20 patients investigated with ^18^F-FLT, 9 had a peripheral volume range of 0%–20%, 5 had a peripheral volume range of 20%–40%, and 6 had a peripheral volume greater than 40%. For CBV, 17 of 31 patients had a peripheral volume range of 0%–20%, 10 had a peripheral volume range of 20%–40%, and 4 had a peripheral volume greater than 40%. For ^18^F-FMISO, 10 of 20 had a peripheral volume range of 0%–20%; 7 had a peripheral volume range of 20%–40%, and 3 had a peripheral volume greater than 40%.

### Analyses of the Peripheral Hot Spots Outside the CE Region

**FIGURE 4.**
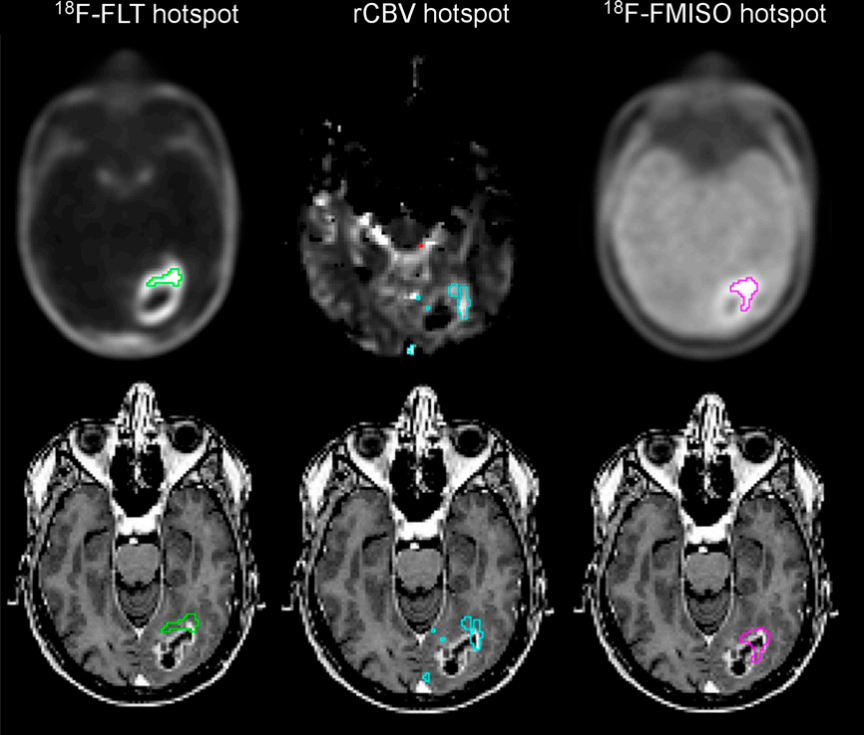
Example of proliferative hot spot in green, hypervascularized hot spot in blue, and hypoxic hot spot in pink on segmented (top) and overlaid (bottom) T1w-Gd MR images.

Considering the strong intratumor heterogeneity observed on multiparametric imaging, we were interested in further identifying subvolumes in the tumor that were likely associated with resistance and early recurrence. Hyperproliferative, hypervascularized, and severely hypoxic hot spots were thresholded (Fig. 4, top) and overlaid on the T1w-Gd images (Fig. 4, bottom). In this example, a percentage of the hyperproliferative region (18%), the hypervascular region (11%), and the most hypoxic region (3%) were located outside the CE region.

**FIGURE 5.**
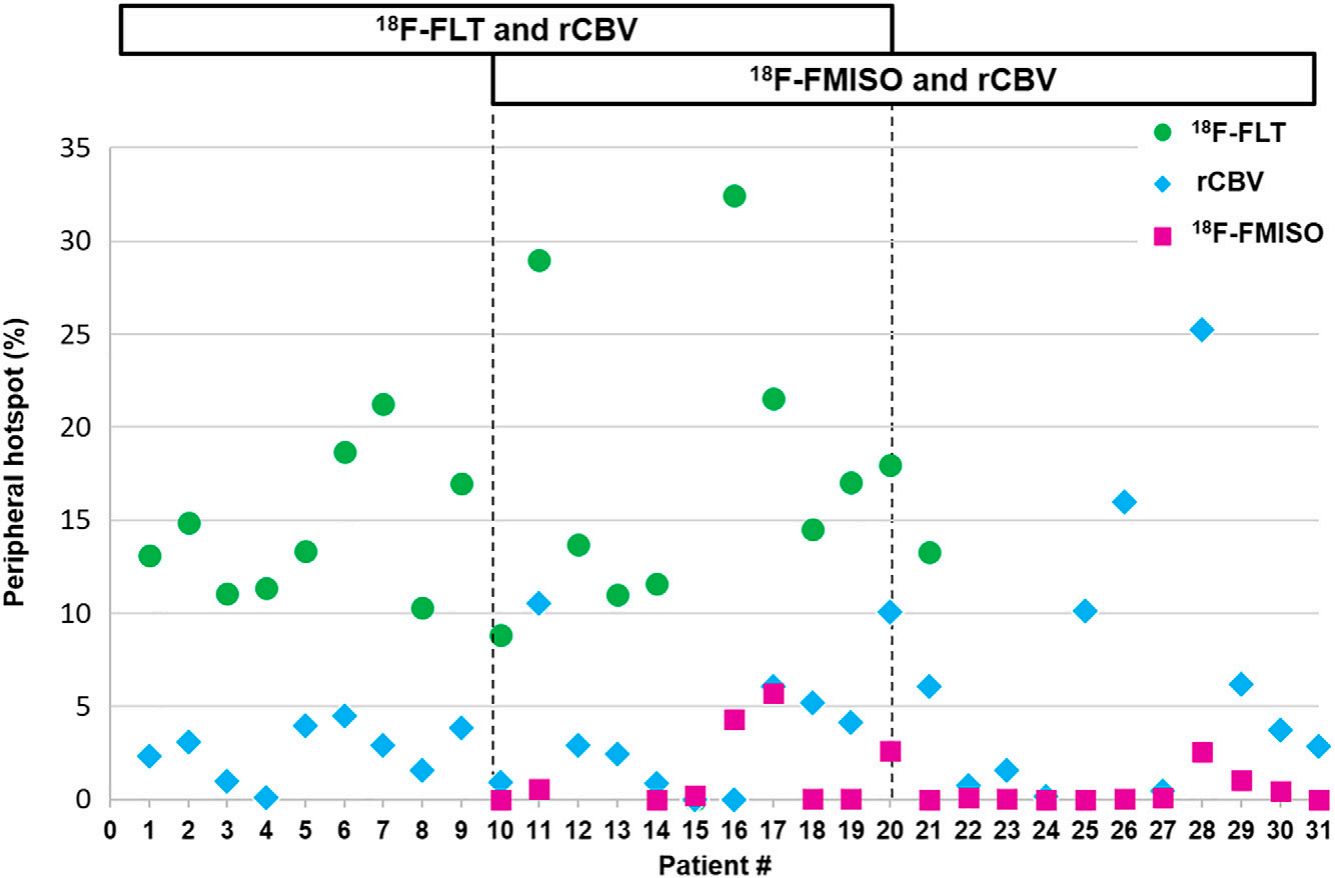
Percentage of peripheral hot-spot volume of ^18^F-FLT, rCBV, and ^18^F-FMISO outside CE volume.

The peripheral hot spots outside the CE volume were calculated for each patient (Fig. 5) and showed that all patients had a hyperproliferative volume outside the CE volume (8.8%–32.5%). More precisely, 1 of 20 had less than 10%, 15 had 10%–20%, and 4 had 20%–40%. Concerning hypervascularized hot spots (0%–25.2%), in 23 of 31 patients the hot-spot fraction was less than 5%, in 7 it was 5%–20%, and in 1 it was 25%. Last, most hypoxic areas were detected mainly in the CE region (0%–5.7%); 14 of 20 patients had less than 1% of the ^18^F-FMISO hot spot outside the CE volume, and the others had around 5% outside the CE volume.

## DISCUSSION

In the context of glioblastoma, intertumoral and intratumoral heterogeneity has been attributed to the failure of standardized treatments. Among the factors influencing tumor growth, the in vivo relationship between proliferation, angiogenesis, and hypoxia remains of great interest relative to the conventionally aggressive region defined on CE MRI. In the context of glioblastoma, the present study is the first one, to our knowledge, to show the spatial distribution of each modality together and relative to CE.

The literature and multivariate analyses have shown that each parameter is independently associated with tumor volume (17,26,36). In gliomas, elevated ^18^F-FLT uptake has been shown to correlate with Ki-67 immunostaining expression and to reflect proliferation (37–39). Also in gliomas, high hypoxia has been shown to be a factor in a poor prognosis (40).

As exemplified in Figure 1, our results confirm that all 3 analyzed parameters are interlinked and that an increase in each parameter occurs concomitantly (9,11). Increased rCBV along with hypoxia might indicate tumor-induced angiogenesis to counteract changes in oxygenation that occur along with the metabolic demand of proliferating cells.

After a visual inspection, each modality in Figure 1 clearly showed a variable uptake distribution that would need to be exploited. Our findings confirm previous publications (9,10,17) indicating that the heterogeneity can be mapped using multimodal imaging.

However, our results on peripheral volume also showed that active tumor tissues were already present in areas that could be considered nonpathologic according to CE on MRI and that therefore might not be targeted by the treatment. This possibility is especially true for ^18^F-FLT PET, which clearly showed that proliferating cells extended outside the CE region on T1w-Gd images, as demonstrated in earlier publications (36).

One of our main results is that the ^18^F-FLT PET volume was greater than the other volumes. The spatial analysis showed that the ^18^F-FLT volume encompasses the rCBV, the CE volume, and the ^18^F-FMISO volume for most patients. This result is in line with previously published results, which also demonstrated that in most cases, the volume of ^18^F-FLT uptake was larger than the tumor volume assessed by anatomic MRI. In glioma, elevated ^18^F-FLT uptake correlates with Ki-67 and reflects proliferation (37,38). This result strengthens the hypothesis that tumoral proliferation is the driving force of the other parameters analyzed in this study, namely angiogenesis and hypoxia.

Various papers have discussed the dependency of ^18^F-FLT uptake on the integrity of the BBB (41). It is recognized that a major limiting factor in ^18^F-FLT uptake is the transport mechanism, and leakage via the disrupted blood–tumor barrier could result in increased uptake. A paper from Watkins et al. (42) suggests that the presence of only a small number of glioma cells could be sufficient to damage the integrity of the BBB, potentially explaining the ability of ^18^F-FLT to detect proliferating cells in nonenhancing regions of the tumor.

The hot-spot analysis showed that all tumors had a hyperproliferative area that extended outside the CE volume, whereas hypervascularized or severely hypoxic areas were mostly included within the CE volume. This result concurs with a recent publication using ^11^C-methionine and demonstrating the presence of metabolic tumor volume after gross tumor resection (43).

These results strengthen the fact that tumor cells have already infiltrated the nonenhancing tissue and ought to be included in the surgical treatment or in the definition of the biologic target volume for radiotherapy (43).

The current standard surgical treatment for glioblastoma is removal of the CE area (44). Because our study showed that metabolically active areas are visible outside the CE volume, removal of only the CE volume could contribute to explaining a rapid recurrence of glioblastoma. We suggest that glioblastoma be resected beyond the CE volume up to the functional limit required to preserve the quality of life (45). The presence of metabolically active areas outside the CE volume may also be used as a parameter for improving the accuracy of the biopsy analysis, and if biopsy and imaging concur, it could be used to improve the quality of resection.

The presence of metabolically active areas outside the CE volume contributes to the definition of gross target volumes for radiotherapy, integrating these findings in the concept of biologic target volume (46). The integration of the metabolically active areas could lead to better tumor control, as it is known that most relapses occur within the irradiation field (47,48) because of radiation resistance in some areas within the irradiated volume. It is assumed that the current radiotherapy regimen does not guarantee the curative doses necessary to counteract radioresistance in some areas of the tumor identified as hot spots in this paper and that this problem may contribute to failure of conventional treatments (49). Radiotherapy is likely to be optimized by specifically targeting these unfavorable biologic characteristics (49,50).

This study had some limitations. We studied each modality only with respect to T1 CE, and we did not perform voxelwise analyses between the various modalities. However, the main goal of the present analysis was to make the study as simple as possible relative to T1 CE in order to provide information the physician will find useful in adapting or tuning therapeutic strategies at the individual level. The use of other types of PET tracer, such as amino acid tracers (^11^C-methionine, *O*-(2-^18^F-fluoroethyl)-l-tyrosine, and 3,4-dihydroxy-6-^18^F-fluoro-l-phenylalanine), might also provide accurate information in mapping regions potentially involved in tumor recurrence. For the hot-spot study, given the method of calculation for each patient, a potential overinterpretation of low activity could occur. As a consequence, comparison of our results to include the peripheral volumes or the hot-spot subvolumes in therapeutic strategies or in stereotactic biopsies would also be of great importance. We are now incorporating this strategy in ongoing clinical trials.

## CONCLUSION

Even if it is difficult to draw a general overview for each individual patient, this study underlines the complementary value of using different multiparametric imaging methods to assess tumor heterogeneity and to define tumor volumes and subvolumes that are likely to be resistant to conventional therapies.

## DISCLOSURE

Funding was received from Conseil Régional de Normandie, Elen Fund, Institut National du Cancer (grant RECF1475), and Agence Nationale de la Recherche-Labex IRON (ANR-11-LABX-0018-01). No other potential conflict of interest relevant to this article was reported.

**KEY POINTS**

**QUESTION:** What is the spatial relationship between proliferation, vascularization, and hypoxia in preoperative glioblastoma patients, with respect to the CE area on T1w-Gd images?
**PERTINENT FINDINGS:** Clinical trials demonstrated the heterogeneity of the 3 parameters measured—namely proliferation, vasculature, and hypoxia—over the classically used CE volume.
**IMPLICATIONS FOR PATIENT CARE:** Incorporating more functional parameters for patient management will improve the delineation of aggressive areas for tumor resection and will help in designing biologic target volume for radiotherapy.
